# Antioxidant and Anti-Inflammatory Effects of *Citrus* Flavonoid Hesperetin: Special Focus on Neurological Disorders

**DOI:** 10.3390/antiox9070609

**Published:** 2020-07-10

**Authors:** Amjad Khan, Muhammad Ikram, Jong Ryeal Hahm, Myeong Ok Kim

**Affiliations:** 1 Division of Life Sciences and Applied Life Science (BK 21plus), College of Natural Science, Gyeongsang National University, Jinju 52828, Korea; Amjadkhan@gnu.ac.kr (A.K.); qazafi417@gnu.ac.kr (M.I.); 2 Division of Endocrinology and Metabolism, Department of Internal Medicine, Gyeongsang National University Hospital and Institute of Health Sciences and Department of Internal Medicine, College of Medicine, Gyeongsang National University, Jinju 52828, Korea; jrhahm@gnu.ac.kr

**Keywords:** hesperetin, neurodegeneration, neuroprotection, Alzheimer’s disease, Parkinson’s disease

## Abstract

Neurodegenerative disorders have emerged as a serious health issue in the current era. The most common neurodegenerative disorders are Alzheimer’s disease (AD), Parkinson’s disease, multiple sclerosis, and amyotrophic lateral sclerosis (ALS). These diseases involve progressive impairment of neurodegeneration and memory impairment. A wide range of compounds have been identified as potential neuroprotective agents against different models of neurodegeneration both in vivo and in vitro. Hesperetin, a flavanone class of citrus flavonoid, is a derivative of hesperidin found in citrus fruits such as oranges, grapes, and lemons. It has been extensively reported that hesperetin exerts neuroprotective effects in experimental models of neurodegenerative diseases. In this systematic review, we have compiled all the studies conducted on hesperetin in both in vivo and in vitro models of neurodegeneration. Here, we have used an approach to lessen the bias in each study, providing a least biased, broad understanding of findings and impartial conclusions of the strength of evidence and the reliability of findings. In this review, we collected different papers from a wide range of journals describing the beneficial effects of hesperetin on animal models of neurodegeneration. Our results demonstrated consistent neuroprotective effects of hesperetin against different models of neurodegeneration. In addition, we have summarized its underlying mechanisms. This study provides the foundations for future studies and recommendations of further mechanistic approaches to conduct preclinical studies on hesperetin in different models.

## 1. Introduction

With current advances in science and technology, the world is extensively experiencing a smooth rise in life expectancy and an associated increase in the incidence of neurodegenerative disorders. Furthermore, neurodegenerative conditions mostly occur in later stages of life; the number of individuals with disorders like Alzheimer’s disease (AD), Parkinson’s disease, and multiple sclerosis (MS) is expected to go higher in the upcoming years. Although the incidences of these disorders are increasing, unfortunately developing therapeutic strategies for treating the neurodegenerative conditions is challenging, and the current management of neurodegeneration remains largely unsuccessful [1]. The neurodegenerative disorders share a multi-faceted set of mechanisms that aid in the progression of these diseases. These factors comprise elevated nitrosative and oxidative stress, excitotoxicity, disturbed calcium homeostasis, inflammation within the brain regions, and interruptions in protein homeostasis that may induce the death of neuronal populations within the brain. The loss of these neuronal cells and its associated signaling leads to the progression of the synaptic and cognitive dysfunction that is naturally associated with major neurodegenerative diseases such as Parkinson’s disease and Alzheimer’s disease.

**Figure 1:**
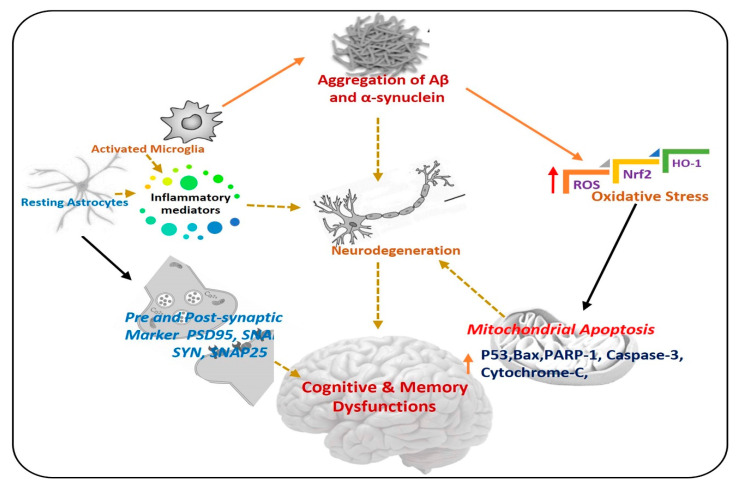
Pathogenesis of neurodegenerative diseases. A diagram showing the role of activated astrocytes, microglia, neuroinflammation, and elevated oxidative stress in the procession of neurodegeneration. Aβ: Amyloid beta, ROS: Reactive oxygen species, Nrf2: Nuclear factor-2 related factor-2, HO-1: Heme-oxygenase-1, PSD-95: postsynaptic density protein 95, SNAP25: Synaptosome Associated Protein 25, Syn: Syntaxin.

The term neurodegeneration is a complex multifactorial process that causes the progressive loss of structure and functions of neuronal cells in the brain and spinal cord, which further causes neuronal dysfunction and memory impairment [2,3]. Neurodegeneration is used for several neurological disorders such as Alzheimer’s disease, Parkinson’s disease, Huntington’s disease, and amyotrophic lateral sclerosis. There are various pathophysiological bases of neurodegenerative diseases but the most important are: accumulation of abnormal proteins, oxidative stress, neuroinflammation, and mitochondrial dysfunction [4,5], in Figure 1, a simple diagram has shown the progression of neurodegenerative diseases, and the role of different factors.

Oxidative stress and neuroinflammation are the initiators of neurodegenerative disorders. Oxidative stress is produced from various factors like the accumulation of abnormal protein, disturbance in the balance of peroxidation and polyunsaturated fatty acids, and high trafficking of Ca^2+^ across the neurons [6]. Elevated oxidative stress is responsible for the activation of several biochemical pathways that induce oxidative damage of other molecules like lipids, proteins, and DNA, leading to neuronal cells death and neurodegeneration [7,8]. Neuroinflammation is inflammation of the central nervous system (CNS), and is due to a complex immune response of the brain to injury, leading to the activation of glial cells and release of inflammatory cytokines, causing serious consequences related to neurodegenerative diseases [9].

Recently, greater attention has been given to phytonutrients obtained from different plant sources, to boost the immune system and counteract the progression of neurodegeneration in different experimental settings. Among the phytonutrients, the flavonoids are a diverse group of natural compounds, which has been extensively studied for the treatment and prevention of various neurological diseases [10]. The word flavonoid is taken from the Latin word meaning yellow, as they have yellow color. Flavonoids are polyphenolic secondary metabolites, occurring naturally in vegetables, herbs, fruits, different types of grains, and some beverages [11,12]. Flavonoids are found in fruits and vegetables with a variable phenolic structure. The flavonoids, soused in various pharmaceutical and medical supplements [13,14], have many beneficial effects on health, including their anti-inflammatory, anti-apoptotic and anti-oxidant effects. They have the ability to scavenge reactive oxygen species (ROS) and activate enzymes that are anti-oxidant in nature, and they can reduce the concentration of substances that have an active role in producing ROS [15,16,17].

Hesperetin, an important bioactive compound in Chinese traditional medicine, has antioxidant and anticarcinogenic properties. Hesperetin is found in abundance in orange and grape juices (200–590 mg L^−1^) consumed in the daily diet [18]. Both Hsd and its aglycone hesperetin have shown various biological activities. For example, hesperetin possesses vitamin-like activity and can decrease capillary permeability (vitamin P), leakiness, and fragility [19]. Currently, it has been indicated that hesperetin confers marked antioxidant, anti-inflammatory, and neuroprotective effects in different models of neurodegeneration [12,20]. The current review is a comprehensive review covering the current research progress on the role of hesperetin in the management of different diseases, with special emphasis on neurodegenerative conditions.

## 2. Methods

### 2.1. Search Methods

We extensively reviewed the research articles showing the beneficial effects of hesperetin in different models of neurodegeneration. The studies were searched from several databases: such as Google Scholar and PubMed. We used different keywords, such as “neuroprotective”, neuroprotection”, and “hesperetin” for the search. The reviewer (Amjad Khan) collected the studies by studying the abstracts of the collected articles.

### 2.2. Inclusion and Exclusion Criteria

a. Studies conducted on any species, age, and sex were included. b. Studies where a comparison between different groups was given (e.g., control group, diseased group, and treated with hesperetin group), where the control mice had administered a physiological placebo/saline or a similar vehicle, were included. Administration of drugs, administration route, and treatment schedule were not considered. No duplicate references, incomplete data, and review articles were included. All studies where the effects of hesperetin on animal models of neurodegeneration were included.

## 3. Chemical Structure, Bioavailability and Blood–Brain Barrier Permeability of Flavonoids

**Figure 2:**
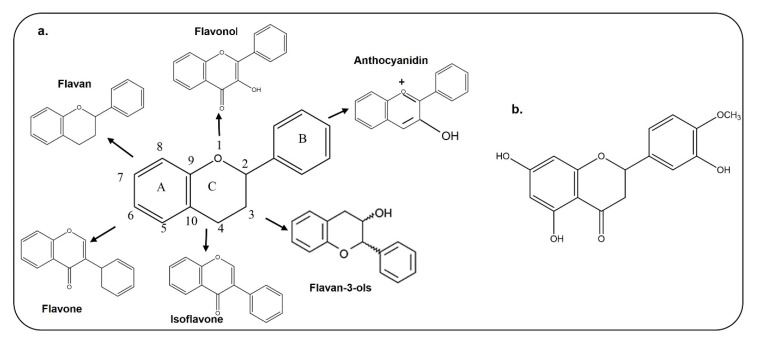
Chemical Structures of different types of flavonoids and hesperetin. **a**. Chemical structure of flavan, flavonol, flavone, isoflavone, flavon-3-ols, and anthocyanidin. **b**. Chemical structure of hesperetin.

The basic structure of flavonoids contains 15 carbon atoms with two benzene rings, which are attached by three carbon atoms (Figure 2); they are classified as flavones, isoflavones, and anthocyanin [21,22]. Chemically, hesperetin is a trihydroxyflavone with three hydroxy groups located at positions 3, 5, and 7, with an additional methoxy substitute also present at position 4 [23]. As interest is growing in the use of dietary flavonoids to combat the oxidative stress-mediated neurodegeneration in CNS-associated pathophysiological processes, including Alzheimer’s disease and Parkinson’s disease, there is a growing concern regarding its entry into the CNS and penetration through the blood–brain barrier. Before absorption from the gut, flavonoids must be released from plant sources by chewing or by the action of the digestive enzymes in the gastrointestinal tract [24,25]. After ingestion, the absorption of flavonoids depends on its physicochemical properties such as molecular size, lipophilicity, solubility, and pKa values [25]. Several studies have analyzed the penetration of flavonoids through the BBB, which has added value to the studies conducted so far. Here, in the case of hesperetin, some studies have been conducted in animals and brain endothelial cells, which showed that citrus flavonoids like hesperetin, naringenin, dietary anthocyanin and polyphenols are taken up by brain cells [26,27]. Studies conducted on rats and pigs have shown that anthocyanin can pass through the blood–brain barrier in these animal models [28]. Another study has also shown that quercetin, kaempferol, and isorhamnetin are detected in the brain of rats after ingestion of gingko biloba extract [29]. The main flavonoid of green tea is epigallocatechin-3-gallate, which, when it was intraperitoneally injected into rats, reached the rat brains [30]. The current in vitro and in vivo studies have shown that flavonoids can cross the BBB and reach the brain to produce its effects. A more comprehensive study related to the absorption, metabolism and execution of its effects has been summarized in a review article [31].

## 4. Flavonoids and Neuroprotection

**Figure 3:**
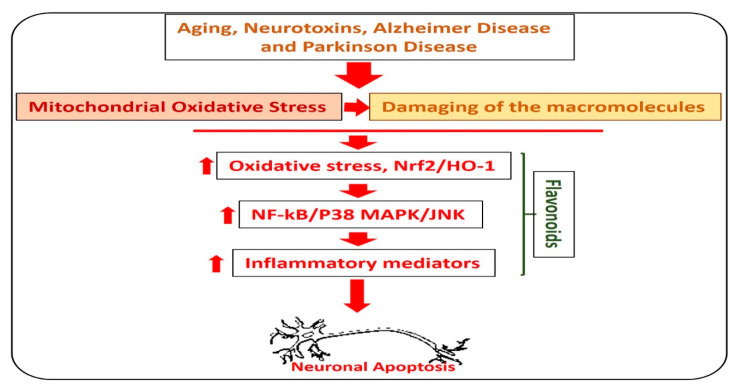
Role of flavonoids in the management of neurodegenerative diseases. A diagram showing the role of hesperetin in the management of neurodegenerative diseases. The image highlights the role of flavonoids against aging, Parkinson’s disease, and Alzheimer’s disease-induced neurodegenerative conditions. As with neurodegeneration, there is an elevation in the level of reactive oxygen species, which facilitates the neuroinflammation by activating the NF-kB and MAP kinases, and release of inflammatory cytokines and neurodegeneration. Interestingly, these effects were markedly reduced with the administration of flavonoids.

Alzheimer’s disease is the most common type of dementia, where, out of 6.8 million people diagnosed with dementia, 5 million had Alzheimer’s disease. In AD, there is an abnormal production of amyloid-beta (Aβ) in the brain, which leads to memory and cognitive dysfunction. The accumulation of Aβ leads to elevation of oxidative stress, neuroinflammation, and neurodegeneration [32]. Currently, there is no effective treatment available for the treatment and prevention of AD [33]. Flavonoids are natural anti-oxidants, which reduce the oxidative stress (Nrf2, HO-1) and amyloid-beta burden (Aβ and BACE-1) in the animal models of AD [34,35]. Quercetin, rutin, silibinin, naringin, hesperidin, and the anthocyanins are the most studied flavonoids that are used to reduce the neuroinflammation and oxidative stress in various types of neurodegenerative diseases [36]. In Figure 3, we show the effects of flavonoids against different types of neurodegenerative diseases.

Similarly, Parkinson’s disease (PD) is the most common neurodegenerative disorder after Alzheimer’s disease, and occurs due to the loss of dopaminergic neurons in the substantia nigra resulting in a decrease in the production of dopamine in the striatum and, subsequently, motor dysfunction [37]. The exact cause of Parkinson’s disease is unknown but it occurs due to the accumulation of abnormal proteins, oxidative stress, environmental toxins, and accumulation of α-synuclein [38]. Several studies have shown the rescuing effects of flavonoids against Parkinson’s disease models, both in vivo and in vitro, where it has been shown that flavonoids reduce oxidative stress and neuroinflammation, and inhibit the formation of α-synuclein. For example, baicalein has shown to decrease the elevated level of α-synuclein by induction of autophagic flux in a rat model of PD [39,40]. In addition, berries containing a variety of flavonoids have shown beneficial effects against animals and cellular models of PD [41].

Huntington’s disease (HD) is another neurodegenerative disease, characterized by psychiatric disturbances, involuntary movements, dementia, and cognitive impairments. Genetically it is associated with the expansion of cytosine adenine guanine trinucleotide repeats in the Huntingtin gene [41,42]. There is no treatment available for the management of HD, although in pre-clinical studies several flavonoids have shown promising protective effects against HD, e.g., hesperidin and naringin in a rat model of HD [43]. Other studies conducted on quercetin, rutin, and myricetin have also reduced the symptoms of HD in animal models of the disease [44,45].

Amyotrophic lateral sclerosis (ALS) is another neurodegenerative disease that targets motor neurons, and whose pathophysiological basis is unknown. Numerous studies attempting to define the pathogenesis of ALS have identified several molecular pathways leading to motor neuron degeneration, which include oxidative stress, glutamate excitotoxicity, apoptosis, abnormal neurofilament function, protein misfolding and subsequent aggregation, impairment of RNA processing, defects in axonal transport, changes in endosomal trafficking, increased inflammation, and mitochondrial dysfunction [46,47]. Similarly, the administration of ginkgo biloba extract has shown promising effects in a transgenic mouse model of ALS, where it improved motor performance and increased survival time [48]. Genistein is a dietary flavone, which has also shown promising therapeutic effects in a mice model of ALS [49]. The above lines of evidence have shown that flavonoids may confer neuroprotection, by regulating multiple aspects of neurodegeneration.

## 5. Citrus Flavonoids: Hesperetin, Dosage and Route of Administration

Flavonoids that are present in citrus fruits like oranges, grapefruits, limes, mandarins, pomelos, and bergamots, are called citrus flavonoids. The most important flavonoids that are found in citrus fruits are naringin, naringenin, quercetin, diosmin, rutin, and hesperetin [50,51]. The citrus flavonoids have shown several beneficial effects including anti-oxidant and anti-inflammatory properties and anti-apoptotic effects. Hesperetin is one of the citrus flavonoids found mainly in the juices, which have been suggested to possess a wide range of pharmacological effects [12]. Hesperetin has been used both orally and intraperitoneally. There is a wide variation in the dosing frequency, but the majority of authors have used it for 4 weeks at a dose of 50 mg/kg/day [12,20].

### 5.1. Absorption, Distribution, and Metabolism of Hesperetin

After extraction, the other main factors that aid in the efficacy of bioactive compounds is their absorption, distribution, and metabolism. It has been indicated that after 20 min of administration of hesperetin, it is converted to hesperetin 7-O-glycoside by an enzymatic reaction. Here, the flavone aglycone is hydrolyzed by beta-glucosidase in the small intestine or colon [52]. A study conducted on humans has shown that the peak plasma concentration of hesperetin is markedly enhanced after the oral intake of orange and grapefruit juices [53]. In regards to blood–brain barrier (BBB) permeability, it has been suggested that hesperetin reaches the CNS, where it may exert neuroprotective effects by counteracting the free radicals generated during cellular metabolism [54].

### 5.2. Neuroprotective Effects of Hesperetin in Neurodegenerative Diseases

The neuroprotective effects of hesperetin have been extensively highlighted in different models of neurodegenerative disease. Some of the main effects of hesperetin are covered here.

#### 5.2.1. Effects of Hesperetin against Alzheimer’s Disease

**Figure 4:**
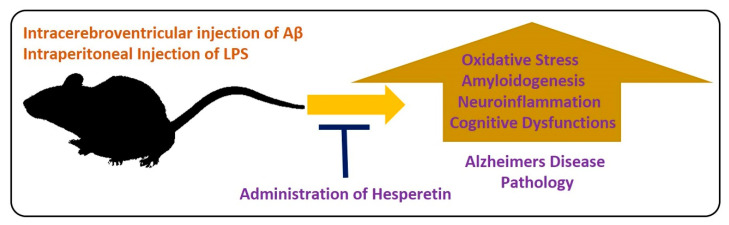
Role of hesperetin in the management of AD-like pathological changes in mice brains. A simple illustration showing the effects of Aβ and LPS on the mice’s brains. The injection of Aβ and LPS into the mice by intracerebroventricular or intraperitoneal injection hesperetin induces oxidative stress, amyloidogenesis, neuroinflammation, and cognitive dysfunction in the mice brains. These effects were markedly reduced with the administration of hesperetin.

For the evaluation of the neuroprotective effects of hesperetin in animal models, different models have been developed. In one study, the effects of hesperetin were analyzed in a rat model of AD. The authors concluded that hesperetin and hesperetin nanoparticles at a dose of 10 and 20 mg/kg for 3 weeks significantly improved the learning and cognitive impairments by reducing the elevated oxidative stress. Moreover, they suggested that the nanoparticles of hesperetin are more effective than simple powdered hesperetin [55]. Similarly, another study conducted by our group evaluated the effects of hesperetin against amyloid beta-induced AD. According to our collective findings, hesperetin significantly reduced the oxidative stress-mediated neuroinflammation, apoptotic cell death, and neurodegeneration. We targeted the endogenous anti-oxidant mechanisms, TLR4-mediated glial cell-mediated neuroinflammation and neurodegeneration. In addition, our findings suggested that hesperetin reduced the cognitive and memory dysfunction in mice, as analyzed by the Morris Water Maze test and Y-maze test [37]. Neuroinflammation is the main inducer of AD [56], so inflammation-mediated neurodegenerative disease models have been extensively used in different experimental settings. LPS is a biomolecule present in the outer membrane of Gram-negative bacteria. LPS targets the toll-like receptor (TLR) 4, and activation of transcription factors, which, in turn, induce a series of inflammatory genes and mediators [57]. Using the LPS-induced neurodegeneration mouse model of AD, we evaluated the effects of hesperetin. Our findings supported the notion that hesperetin significantly reduced LPS-induced neurodegeneration and memory impairment. Figure 4 depicts the hypothesis that hesperetin reduces toxin-induced neuroinflammation and neurodegeneration.

#### 5.2.2. Effects of Hesperetin against Parkinson’s Disease

**Figure 5:**
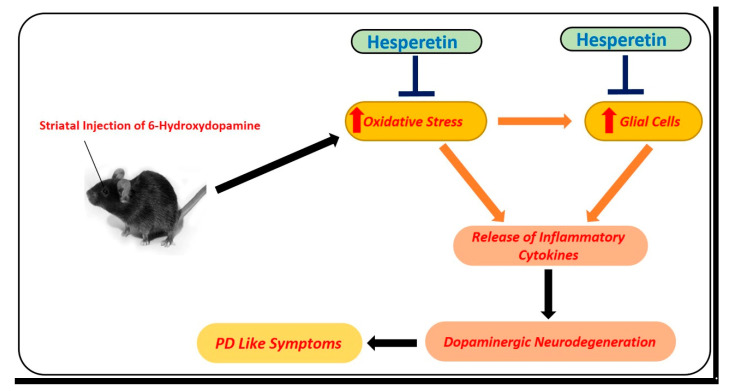
Role of hesperetin in the management of PD-like changes in the mice brains. A simple diagram showing the effects of 6-hydroxydopamine, a potent and known environmental neurotoxin that is used for modeling of PD-like symptoms in mice. Unilateral injection of 6-hydroxydopamine into the striatum of the mice induced oxidative stress, neuroinflammation, and motor dysfunction in mice. These effects were markedly reduced with the administration of hesperetin.

Parkinson’s disease is another devastating neurodegenerative disease. The pathophysiology of PD involves the loss of dopaminergic neurons in the substantia nigra, which leads to motor and cognitive dysfunction. 6-hydroxydopamine (6-OHDA) is an environmental neurotoxin that is used for the induction of PD–like symptoms in animals. To analyze the effects of hesperetin against PD-like conditions, hesperetin has been used at a dose of 50 mg/kg for one week. According to the findings, hesperetin reduced oxidative stress by regulating the transcription factor Nrf2, neuroinflammation (NF-kB), and apoptotic cell loss (mitochondrial apoptosis). Moreover, they showed that hesperetin markedly reduced the motor dysfunction in the 6-OHDA-induced PD rats [58] (Figure 5).

#### 5.2.3. Effects of Hesperetin against Temporal Lobe Seizures

Another very common neurodegenerative condition is known as temporal lobe epilepsy, also called temporal lobe seizure. Temporal lobe seizure is often called focal seizures with decreased awareness. Here, the individual remains aware of what is happening, but during intense conditions, the patient is unresponsive. The lips and hands may become paralyzed. Several studies have suggested that repeated seizures affect cognitive function, including executive functions, intelligence, judgment, attention, and problem solving [59]. Kainic acid is used to induce epilepsy in the mouse. The main pathogenesis of kainic acid is to induce seizures and neuroinflammation. Dose-dependent oral administration of hesperetin (5, 10, or 20 mg/kg/day) delayed the onset of a seizure by inhibiting the proinflammatory kinases in the hippocampus of the epileptic mice [54].

#### 5.2.4. Effects of Hesperetin against Ischemic-Reperfusion Injury

Ischemia-reperfusion injury (IRI) is the acceleration of cellular death and loss of function following the reinstatement of blood flow to ischemic cells. Restoration of blood flow is important to heal the ischemic tissues. However, this reperfusion causes more injury, disturbing the normal function, and affects the viability of the organ [60]. IR injury is induced by increasing the intraocular pressure of mice to 110 mmHg for 40 min, which causes ganglionic cell injury, oxidative stress, inflammation, and cell death. To evaluate the effects of hesperetin against ischemia-reperfusion (I/R) injury, the mice were treated with either normal saline (NS, 0.3 mL/day) or with water-soluble hesperetin (0.3 mL, 200 mg/kg/day). According to the reported effects, hesperetin protected ganglion cells from ischemic reperfusion injury by reducing oxidative damage (LPO) and reducing apoptotic cell death (Bax and Caspase-3). In addition, hesperetin reduced the inflammatory effects through the downregulation of microglial cells, such as Iba-1 [61].

#### 5.2.5. Effects of Hesperetin against Cadmium-Induced Neurodegeneration

Cadmium is a heavy metal and environmental pollutant that accumulates in human and animal bodies. The accumulation of cadmium can cause various health hazards and it can affect the permeability of the blood–brain barrier, which causes oxidative stress and neurodegeneration [62,63]. To analyze the effects of hesperetin against cadmium-induced neurodegeneration and memory impairment, the authors administered hesperetin (40 mg/kg for 3 weeks), which reduced oxidative stress, restored mitochondrial dysfunction, reduced apoptosis, and upregulated antioxidant transcription factors in the brains of rats [64]. Chronic administration of hesperetin (five weeks, 10 and 50 mg/kg) showed neuroprotective effects by protecting the mice’s brain against oxidative stress, as revealed by the reduced level of lipid peroxidation, and activation of the endogenous antioxidant defense mechanisms, including the catalases, total SOD and GSH-related enzymes. Moreover, hesperetin did not cause apoptosis in the brain, even at the higher dose. These qualities of hesperetin make it a useful antioxidant and neuroprotective agent in the management of neurodegenerative diseases [65]. 

#### 5.2.6. Neuroprotective Effects of Hesperetin in Cellular Models of Neurodegeneration

To further strengthen the hypothesis that hesperetin protects the brain against neurodegeneration, various studies have analyzed the effects of hesperetin against different models of neurodegeneration in neuronal cell lines, including neuroblastoma SH-SY5Y, PC12 cells, and mouse hippocampal HT22 cells [66]. In a study conducted on neuroblastoma SH-SY5Y cells, where the neuronal injury was induced by hydrogen peroxide (75 μmol/L), interestingly, there was significant protection against the peroxide-induced neuronal damage when treated with hesperetin and 5-nitro-hesperetin at a concentration of 0.01 μmol/L. Similarly, caspase activity (caspase-3 and caspase-9) were significantly decreased with the administration of hesperetin. ERK1 and ERK2 are serine/threonine kinases, which participate in the control of numerous processes including apoptosis, cell proliferation, immune responses, nervous system function, and RNA synthesis and processing. According to the data shown, hesperetin (100–300 nM, 15 min) markedly increased the level of ERK1/2 phosphorylation in a dose-dependent manner [67,68]. H_2_O_2_ causes cytotoxicity in PC12 cells, which induces cell damage, reduces the mitochondrial membrane potential, reduces antioxidant enzymes, such as catalase (CAT) and glutathione peroxidase (GSH-Px), induces the release of cytochrome C into the cytosol, activates caspase-3, induces reactive oxygen species, and depletes glutathione in PC12 cells. Interestingly, these effects were significantly inhibited with the administration of hesperetin [69]. Similarly, to evaluate the effects of hesperetin against a rotenone-induced in vitro Parkinson disease model, human neuroblastoma SH-SY5Y cells were treated with rotenone with or without hesperetin. The findings indicated that hesperetin reversed the elevated oxidative stress and mitochondria dysfunction. Out of seven used flavonoids, hesperetin protected more than 50% of the cells at different concentrations. It was also noted that hesperetin had a medium potency against GSK-3β and no activity against CK-1δ, which needs more comprehensive studies (59).

Similar results were obtained when the H_2_O_2_- and l-glutamate-treated cortical rat neuronal cells were treated with hesperidin and hesperetin. The findings showed that hesperetin is a more effective anti-oxidant than hesperidin [70]. Moreover, when the hippocampal HT22 and murine microglia BV2 cells were co-treated with or without hesperetin and lipopolysaccharide (LPS), the findings suggested that hesperetin significantly reduced the LPS-induced neuroinflammation, as indicated by the representative western blot results of TL4, p-NF-kB, GFAP and Iba-1 [13]. Based on its various pharmacological actions demonstrated in these studies, hesperetin may protect the neuronal cells against neurodegeneration. The in vitro findings support the notion that hesperetin is effective against in vivo and in vitro neurodegenerative disease models.

### 5.3. Anti-oxidative Effects of Hesperetin

Oxidative stress is an imbalanced redox state, showing either excessive production of reactive oxygen species (ROS) or impairment of the antioxidant defense system of the cells. The brain is the most susceptible organ to the effects of ROS, because of its high demand for oxygen and the presence of peroxidation-susceptible lipid cells. Extensive research has highlighted that oxidative stress is playing a crucial role in the progression of neurodegenerative disorders such as Alzheimer’s disease and Parkinson’s disease. Agents that counteract excessive level of ROS are attaining the highest interest in the management of neurodegenerative conditions [71].

Currently, several natural and plant-derived compounds are showing efficacy in the management of neurodegenerative conditions [72]. Hesperetin has shown tremendous antioxidant effects by scavenging the elevated ROS and by boosting the endogenous antioxidant defense mechanisms, specifically by upregulating the expression of transcription factor nuclear factor-2 erythroid-2 (Nrf2) and its downstream target heme oxygenase-1 (HO-1). In a previous study, oxidative stress was induced by H_2_O_2_ in RPE-19 cells and treated by hesperetin. The results showed that hesperetin significantly protected the RPE-19 cells from the elevated oxidative stress, by inhibiting apoptotic cell death (by regulating the expression of Bax, Bcl2, and caspase-3), production of ROS and MDA, and enhancing the expression of SOD and GSH, which may cause the activation of Keap-1, and Nrf2/HO-1 signaling [73]. Similarly, in rats, hyperuricemia was induced by IP (intraperitoneal) injection of potassium oxonate (250 mg/kg), with or without hesperetin (5 mg/kg), and orange juice (5 ml/kg for two weeks). The findings showed that orange juice and hesperetin prevented oxidative stress by boosting the antioxidant mechanism and decreasing lipid peroxidation [74]. Another study induced cataracts in rats by sodium selenite (20 µmol/kg body weight), and then injected the rats with hesperetin. The findings suggested that hesperetin and its derivatives reduced the oxidative stress in the cataract lens, although it had less effects on systemic antioxidant levels [75].

The antioxidant effects of hesperetin (50 mg/kg/day) were also evaluated in lead (500 mg Pb/L)-treated adult rats. The results suggested that hesperetin could minimize the unwanted effects of lead by reducing the oxidative damage, and may play a pivotal role in the management of lead-induced neurotoxicity [76]. Similarly, it has been shown that administration of hesperetin (840 mg/kg day) to cadmium-treated rats (83 mg/kg/day/s.c for 21 days) reduced the levels of LPO, a marker of thiobarbituric acid reactive substances (TBARS) and lipid hydroperoxides (LOOH), and elevated the levels of plasma non-enzymatic antioxidants, such as reduced glutathione (GSH) [77]. The same types of findings were obtained, when hesperetin (10 and 50 mg/kg for 5 weeks) was given to 7,12-dimethylbenz (a) anthracene (DMBA)-treated mice (34 mg/kg BW in corn oil two times a week for 2 weeks). Here, hesperetin markedly reduced the level of lipid peroxidation and protein oxidation, while it enhanced the expression of the antioxidant defense system by increasing the catalases, the SODs, and the GSH/GSSG ratio [78]. When the hesperetin was tested in streptozotocin (STZ)-induced type 1 diabetes mellitus (T1DM), the findings suggested that the level of LPO, GSH, and antioxidant genes (Nrf2 and HO-1) were significantly upregulated with the administration of hesperetin [79]. The overall findings suggest that hesperetin is a strong antioxidant flavonoid, which may reduce elevated oxidative stress, thereby relieving oxidative damage.

### 5.4. Anti-neuroinflammatory Effects of Hesperetin

**Figure 6:**
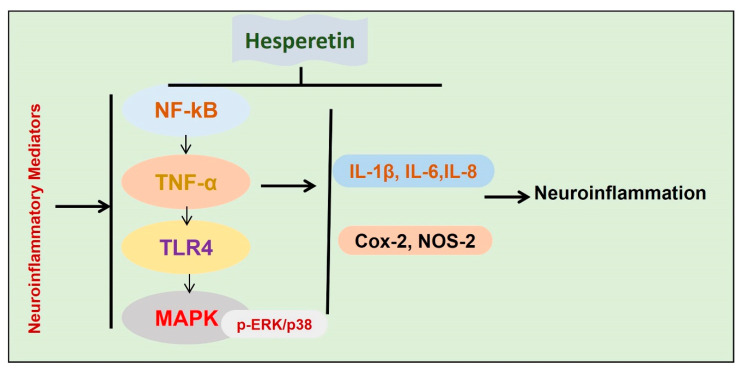
Effects of hesperetin against neuroinflammatory mediators. A diagram showing the effects of hesperetin against the inflammatory mediators in different models of neurodegenerative diseases.

Inflammation is the main contributor to the progression of neurodegenerative conditions [62,80]. In the execution of neuroinflammation, different cells are involved, such as activated microglia astrocytes [80]. To highlight the anti-inflammatory effects of hesperetin, different studies have been conducted so far. In one study, we analyzed the effects of hesperetin against amyloid beta-induced neuroinflammation and neurodegeneration. Collectively our findings have suggested that hesperetin reduces Aβ-induced oxidative stress, thereby reducing activated astrocytes and microglial cells. The suppression of the glial cells was accompanied by reduced phosphorylation of NF-kB, and the release of inflammatory mediators. The rescuing effects of hesperetin against Aβ-induced neuroinflammation were further confirmed with in vitro studies, showing that the inhibition of TLR4 and p-NF-kB by hesperetin was comparable to the specific pharmacological inhibitor p-NF-kB [12]. In another study, we treated the mice with lipopolysaccharide (LPS) and checked the anti-inflammatory effects. The findings suggested that, with the administration of hesperetin, the inflammatory effect was markedly reduced, as indicated by the reduced expression of TLR4, GFAP, Iba-1, and p-NF-kB. We compared the inhibition of TLR and p-NF-kB with their specified pharmacological inhibitors, and the collective findings supported the notion that hesperetin significantly reduced the inflammation induced by LPS [20]. Another study showed potential anti-neuroinflammatory effects of hesperetin against LPS-stimulated BV-2 microglial cells [81]. The authors showed strong inhibition of MAP kinases with the administration of hesperetin, as hesperetin reduced the expression of p-ERK and p38, thereby reducing the level of inflammatory cytokines [82]. Conclusively, the overall findings indicated that hesperetin has potential neuroprotective effects against activated inflammatory mediators in neurodegenerative disease models (Figure 6).

## 6. Conclusions and Future Considerations

Neurodegenerative diseases are caused by a plethora of effectors, such as elevated oxidative stress, neuroinflammation, and apoptotic cell death [82]. The pathophysiology and clinical outcomes of neurodegeneration may be reversed with different types of flavonoids, one of which is hesperetin. The ability of hesperetin to reverse the different inducers of neurodegenerative diseases, including elevated oxidative stress, neuroinflammation, and apoptotic cell death, making hesperetin a candidate drug for the prevention of neurodegenerative conditions. The safety, efficacy, and cheap availability of these citrus flavonoids make them candidate drugs to be further studied and presented for pre-clinical and clinical studies for the management of neurodegenerative disorders. However, the current knowledge regarding the use of hesperetin is quite generalized and needs deeper investigations and research. The translation of animal studies into human studies will need a more comprehensive understanding of hesperetin. Special focus will be given to pharmacodynamic and pharmacokinetic studies. There are still only a few studies available on the usage of hesperetin in the management of neurological disorders. From the available literature, it can be concluded that hesperetin may serve as a candidate drug for the management of neurodegenerative diseases.
